# Translational simulation for rapid transformation of health services, using the example of the COVID-19 pandemic preparation

**DOI:** 10.1186/s41077-020-00127-z

**Published:** 2020-06-03

**Authors:** Victoria Brazil, Belinda Lowe, Leanne Ryan, Rachel Bourke, Clare Scott, Simone Myers, Hellen Kaneko, Jane Schweitzer, Brenton Shanahan

**Affiliations:** 1 Faculty of Health Sciences and Medicine, Bond University, Gold Coast, Australia; 2 Department of Anaesthesia, Gold Coast Hospital and Health Service, Southport, Australia; 3 Simulation Service, Gold Coast Hospital and Health Service, Southport, Australia

**Keywords:** Translational simulation, COVID-19, Pandemic, Healthcare

## Abstract

Healthcare simulation has significant potential for helping health services to deal with the COVID-19 pandemic. Rapid changes to care pathways and processes needed for protection of staff and patients may be facilitated by a translational simulation approach—diagnosing changes needed, developing and testing new processes and then embedding new systems and teamwork through training. However, there are also practical constraints on running in situ simulations during a pandemic—the need for physical distancing, rigorous infection control for manikins and training equipment and awareness of heightened anxiety among simulation participants. We describe our institution’s simulation strategy for COVID-19 preparation and reflect on the lessons learned—for simulation programs and for health services seeking to utilise translational simulation during and beyond the COVID-19 pandemic. We offer practical suggestions for a translational simulation strategy and simulation delivery within pandemic constraints. We also suggest simulation programs develop robust strategies, governance and relationships for managing change within institutions—balancing clinician engagement, systems engineering expertise and the power of translational simulation for diagnosing, testing and embedding changes.

## Introduction

Healthcare simulation is at a cross roads as healthcare professionals, teams and systems deal with the COVID-19 pandemic. Many simulation centres have shut their doors, in line with social distancing rules, and combined with the urgent needs of health services to draw faculty back to the front line. However, simulation services and programs that are ‘truly translational’ [1, 2]—integrated and focused on emerging clinical priorities—are undertaking unprecedented volumes of simulation activity. This article explores our institution’s simulation strategy for COVID-19 preparation and reflects on the lessons learned—for simulation programs and for health services seeking to utilise translational simulation during and beyond the COVID-19 pandemic. We describe our strategy development and context, simulation delivery activities and outcomes and offer principles and practical suggestions for how simulation can directly and rapidly respond to urgent need for health service transformation.

Healthcare simulation offers numerous opportunities for pandemic preparation [3, 4]. Training healthcare professionals for effective use of personal protective equipment (PPE), for new and expanded roles (e.g. critical care skills and procedures) and for public health tasks (swabs, contact training) can be accelerated and perfected using simulation. The structure and skills used for debriefing in healthcare simulation offer guidance for teams using clinical event debriefing to learn and adapt in a rapidly changing environment [5]. Teams are challenged in novel ways—difficulties in communicating due to PPE and isolation rooms, changed procedures and protocols—and simulation can help shape and practice new routines. System level issues, including intra-hospital transfers, team interfaces and re-tooling spaces for new functions (e.g. expanded intensive care capacity) can be addressed using multilayered simulation approaches. More sobering learning objectives may include simulation for communicating with patients and families about end of life and resourcing constraints [6] (using Facetime and wearing PPE) and other palliative care skills.

But simulation may also cause harm during times of crisis. For example, the abrupt introduction of modified airway management simulations to embed COVID-19 changes into an emergency department may create confusion and anxiety if undertaken without clear objectives, clinician leadership and engagement and careful pre-briefing and debriefing.

Turning the promise of simulation into reality for COVID-19 preparation requires a translational approach—a simulation program that is attuned to emerging priorities, has strong relationships with clinicians and service leadership and with the skills and capacity to apply (or develop) simulation strategies to address those issues. Translational simulation describes healthcare simulation focused directly on health service priorities, improving teams and systems through ‘diagnostic’ functions and through iteratively developed simulation-based interventions [1]. In the context of the COVID-19 pandemic, this method offers a ‘rapid prototyping’ approach to reviewing and revising care processes that need significant change to accommodate the need to protect staff from COVID infections.

## Our simulation strategy for COVID-19 preparation

**Fig. 1:**
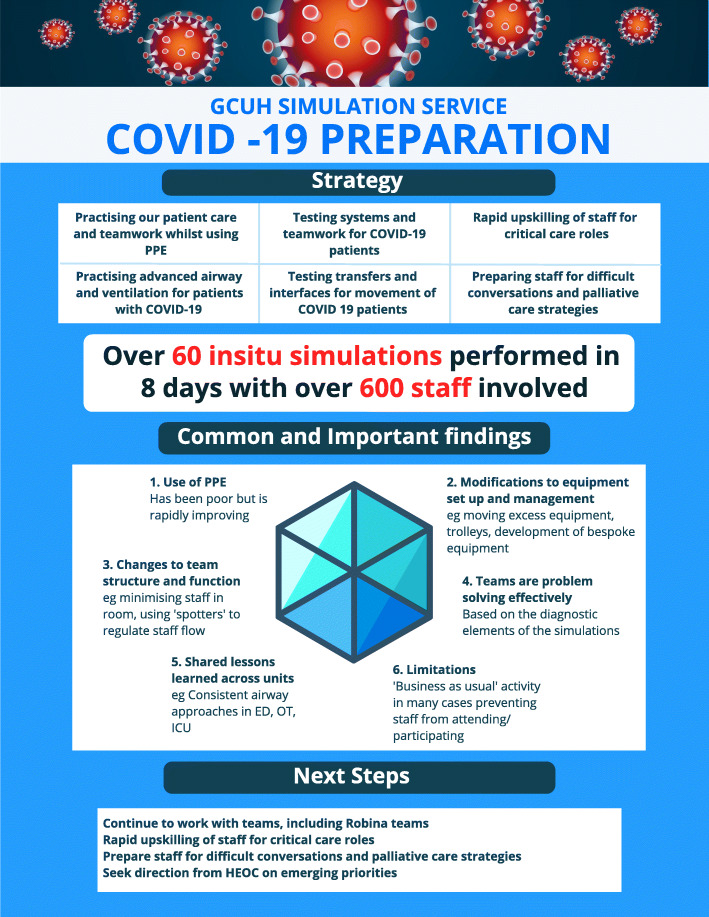
GC Simulation Service report phase 1

On March 8th, 2020, we introduced a simulation strategy for COVID-19 preparation for the Gold Coast Hospital and Health Service (GCHHS). At that time, there were 70 coronavirus cases identified in Australia, but experience in Wuhan and Europe had prompted health services to begin pandemic preparation in earnest. We developed a six-point strategy and initial actions to guide the simulation team approach (Fig. 1).

### Our context and team

The GCHHS is comprised of a number of services and healthcare facilities, including the main Gold Coast University Hospital, an 850-bed tertiary referral hospital which employs over 6000 clinical staff. The GCHHS Simulation Service was established in 2013, focused on developing high performing teams and systems. The program includes educationally focused simulation but extends to ‘in situ’ simulation in clinical areas designed for translational impact—diagnosing and addressing important process and teamwork issues in patient care. Over 200 in situ simulations were run at GCHHS in 2019, through partnership with clinical services wishing to target various quality improvement goals. These services include emergency medicine, maternity, paediatrics, outpatient clinics, cardiac catheter suite, trauma, mental health, operating theatre, stroke services, rehabilitation, Medical Emergency Team (MET) calls and the Afterhours Care Unit. The work of the Simulation Service was originally driven by partnerships within clinical areas in a ‘bottom up’ approach. In 2019, a formal *High-performance Clinical Teamwork Strategy* was endorsed by the GCHHS Board and Executive, in which the Simulation Service is collaborating with the Quality and Safety Unit, Relational Coordination Unit, Professionalism Programs and Bond University Faculty of Health Sciences and Medicine.

The Simulation Service staffing includes three Simulation Educators with nursing and technical expertise, a clinical facilitator (nursing) and a part time medical director. This core group all have had dedicated simulation educator training and collective experience of more than 30 years in healthcare simulation delivery. However, the activity of this group is leveraged by a larger network of simulation educators within the health service, including medical and nursing simulation experts in emergency medicine, anaesthetics, perioperative services, women’s health and other areas. This community of practice has evolved over time and been strengthened through internal faculty development. The faculty development program is conducted three times per year and comprises four sessions of approximately 4 h: designing and delivering simulation, technical aspects of simulation delivery, debriefing and a debriefing masterclass. These structured sessions are complemented by subsequent peer support and coaching of attendees by the Simulation Service team, with the aim of enabling departments to deliver simulation autonomously.

### Our strategy development

We anticipated institutional needs for COVID-19 preparation at the individual, team and system level. Given our existing relationships with clinical services, our focus was on team and system challenges. We felt that our specific expertise in building relationships and shaping culture through simulation [7, 8] was likely to be more important than ever as we undertook rapid and urgent high stakes change. Our strategy was aligned with the *Queensland Health Pandemic Influenza Plan* [9] and guided by the local Health Emergency Operations Centre (HEOC), to whom we provided biweekly reports.

The overall approach and specific activities were also informed by a global network of simulation educators, connected through social media, a small amount of published literature and many personal communications [4, 10–12]. The simulation team met 1–2 times per week during this period to review progress and plan next steps.

### Our simulation activity and outcomes

In the 30 days from March 8th, 2020, we delivered more than 250 translational simulations, involving more than 1500 healthcare staff, across multiple hospital departments. This is a greater volume of translational simulation than we delivered in all of 2019.

There were common and important findings in our early experience in working with teams across a range of clinical contexts (Fig. 1). Initially, concerns and uncertainties about PPE dominated many team discussions, and simulation sessions were a chance to inform and practice PPE skills. Individual and teams rapidly improved in this regard with both real and simulated experience. In this early phase, clinicians sought out exposure to simulation sessions with a sense of urgency.

Many clinical care pathways required review and modification to protect staff from droplet, aerosol and contact exposure. Teams needed to adapt to a new balance between the urgent patient care needs and compliance with protective measures. These logical next steps in our simulation activity involved changes to tasks, changes to team structure and function and changes to physical environments and equipment. Examples included as follows:

- Medical Emergency Team response
- Emergency Department intubation
- Endotracheal intubation for elective procedure on operating theatre
- Management of vaginal delivery
- Post-partum haemorrhage management
- Urgent transfer of maternity patient to operating theatre
- Transfers of patients to ICU
- Care of deteriorating patient in interventional radiology and cardiac catheter lab
- Major trauma reception
- Management of acute behavioural disturbance in the emergency department and in psychiatric unit
- Cardiac arrest management

**Fig. 2:**
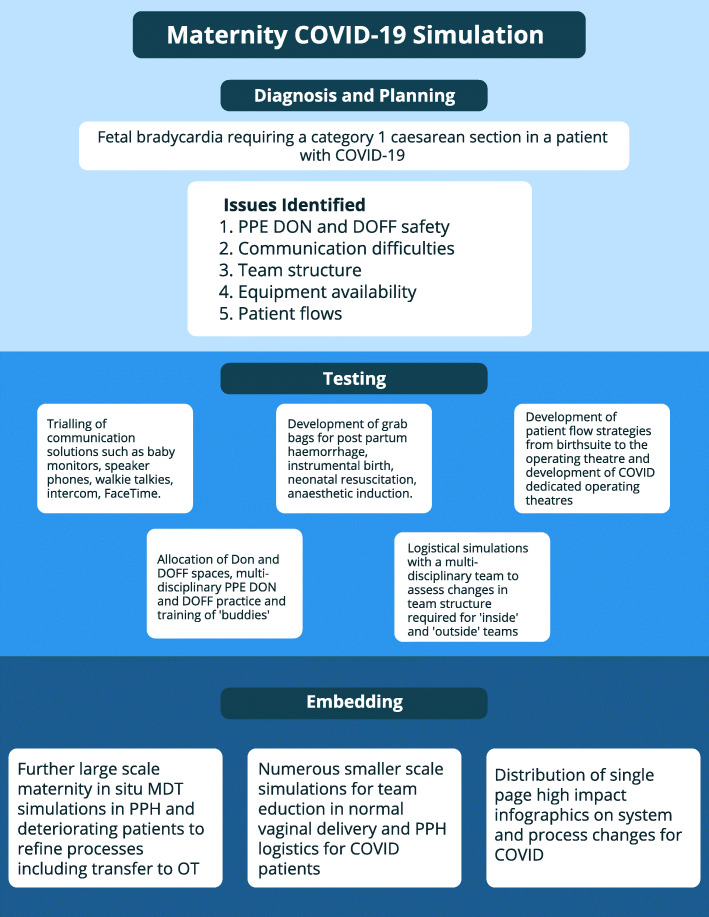
Phased simulation approach in maternity services

Infection Control staff were present for many of the diagnostic phase simulations and provided advice on the application of guidelines in specific, dynamic care contexts. The evolution of one unit’s simulation activity during the 30-day preparation period is illustrated in Fig. 2*.* Although conceptually considered as discrete stages of ‘diagnosis’, ‘testing’ and ‘embedding’, the simulation process was iterative over the 30-day period.

Communication strategies for overcoming the physical barriers between ‘inside teams’ (with patients in isolation rooms, wearing PPE) and ‘outside teams’ (in clean areas, supporting the needs of inside team with variable visual observation of inside team) emerged as the greatest challenge for teams. Walkie talkies, mobile phones, baby monitors and video conference options were all imperfect.

The urgency and priority of pandemic preparation created a high degree of collaboration within and between clinical units. Although participating in simulation activity was initially limited by clinicians busy with ‘business as usual’ care, this changed rapidly once the institution moved to ‘Tier2’ with elective surgery and other non-essential activity (including much of our purely educationally focused simulation work) cancelled.

In the later phases of our preparation, clinical teams were applying lessons from simulation to the real or suspected COVID-19 patient care (e.g. ED intubation, operating theatre flows, maternity care). This provided valuable feedback for the conduct of simulations, as well as iterative improvement in revised clinical care processes, enabled by the close connection of the simulation and clinical teams.

“*Thanks for all the SIMs that have been done - I can say firsthand that they’re very helpful! It would be great if more anaesthetic nurses can get through them as mine hadn’t and so a lot of my cognitive load was going through what we can and can’t do*” (Anaesthetic registrar)

## Lessons for rapidly responding to health service crises using simulation

At 7th April, Australia had 5908 confirmed cases of COVID-19 and a falling rate of new cases each day. At the time of writing, we have not seen the dramatic increase in COVID-19 patient numbers than have occurred in other parts of the world in our health service, and we make no claim as to the ‘success’ of this preparation in terms of patient care or service outcomes.

Our translational simulation service has been able to rapidly increase simulation activity and adapt focus to COVID-19 pandemic preparation. We have increased staff confidence with PPE, rapidly developed new pathways and procedures for patient care, embedded those pathways through various training modalities and increased teams’ confidence in approaching the possible task ahead. We suggest there are lessons for simulation programs to build current and future capacity for responding to a crisis such as the COVID-19 pandemic preparation, based on reflection on the strengths and weaknesses of our approach.

### Develop a clear strategy for using translational simulation when rapid change is required in health services

In the midst of urgent and high stakes change, we were tempted (and did) conduct high volumes of familiar simulation techniques focussed on familiar targets (e.g. in situ simulation for airway management). However, a translational simulation approach requires a guiding strategy—ensuring simulation targets and techniques that are fully integrated with emerging, broad priorities and plans of health services. We developed our strategy based on longstanding formal and informal connections with multiple areas within the health service—at both executive level and with frontline clinicians.

### Build longitudinal relationships between simulation programs and clinical services—to help target high yield simulation activity and to optimise clinician engagement

**Fig. 3:**
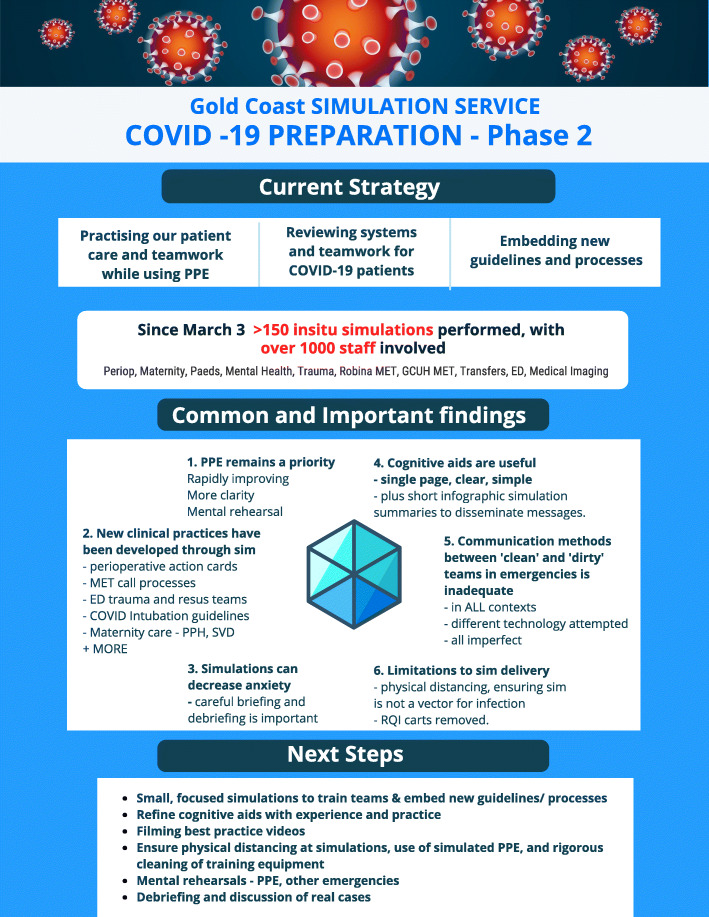
GC Simulation Service update phase 2

Our simulation teams worked with frontline clinicians to undertake urgent, high stakes change processes that would normally take months or years to occur (Fig. 3). Teams were highly motivated and engaged, and previous extensive experience of in situ simulation and working with the Simulation Service allowed early and rapid simulation activity as part of pandemic preparation.

However, our impact was also *limited* by our previous scope and relationships. We gave limited support to those parts of the institution we had not collaborated with before, e.g. patient transfer officers, security staff and non-clinical areas. We had no simulated patient or consumer involvement; only patients and essential staff were allowed within the hospital during this period, and hence there was limited focus on patient experience during this preparation.

### Develop clear lines of communication and responsibility with other units responsible for change management throughout the institution

The Simulation Service had the clinician relationships and simulation skills to rapidly design, update and train staff in new processes. Initially, the simulation team was conceptionally ahead of other teams in the hospital, with a vision for the process adaptations required of teams to accommodate protecting staff from COVID-19. However, this put the simulation team in a precarious position where we were trialling new equipment and processes, based on first principles and emerging literature, but without the usual management approval and protracted evidence-based practice. Governance and oversight of changes were variable—some were approved/ ratified by relevant oversight groups, while others were informally adopted by teams or departments. The boundaries between the Simulation Service supporting and enabling change processes versus taking responsibility for those changes were not always clear.

This dilemma is not new for those healthcare simulation programs that seek to engage in quality improvement activities [13], but the urgency of the COVID-19 preparation exacerbated both the strengths and weaknesses of translational simulation for this purpose. Overall, clinicians and clinical leaders dramatically over-estimated the ability of individuals and teams to adapt to new processes and had limited appreciation of unintended consequences of changes. Our simulation activities revealed inadequacies in lengthy documents that had been produced to guide clinicians for COVID-19 care, in a classic illustration of the gap between ‘work as imagined’ and ‘work as done’ [14].

In an effort to close that gap, the Simulation Service produced videos, infographic summaries and cognitive aids to summarise findings and changes and to help disseminate messages (Additional files 1, 2, 3 and 4).

Our team lacked formal expertise in human factors and systems engineering. Integration of simulation within a more formal user centred design approach [15], including task analysis and ‘desktop’ cognitive walk throughs, may have allowed better targeting of manikin-based live simulations and complemented clinician-led change.

### Develop a wide range of simulation skills and approaches to rapidly adapt to novel simulation objectives

**Fig. 4:**
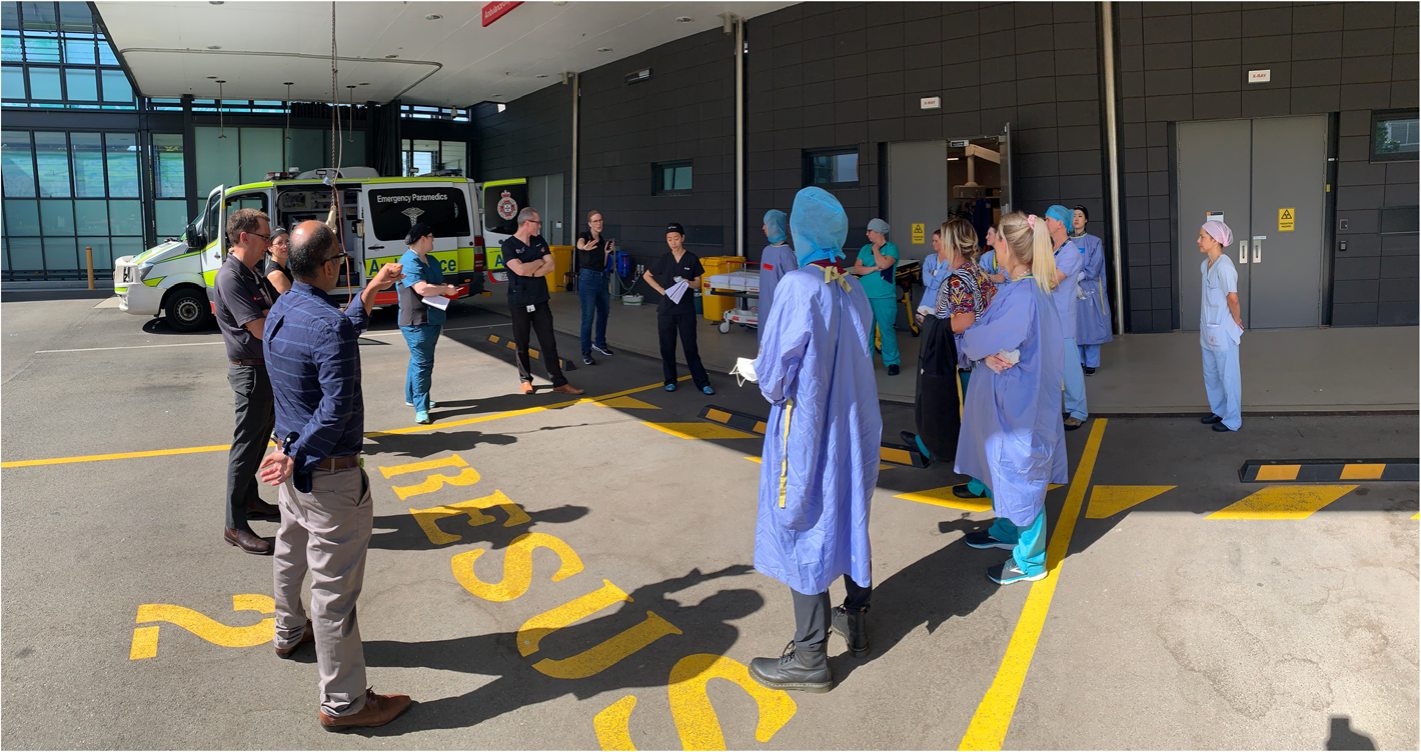
Physical distancing during debrief

**Fig. 5:**
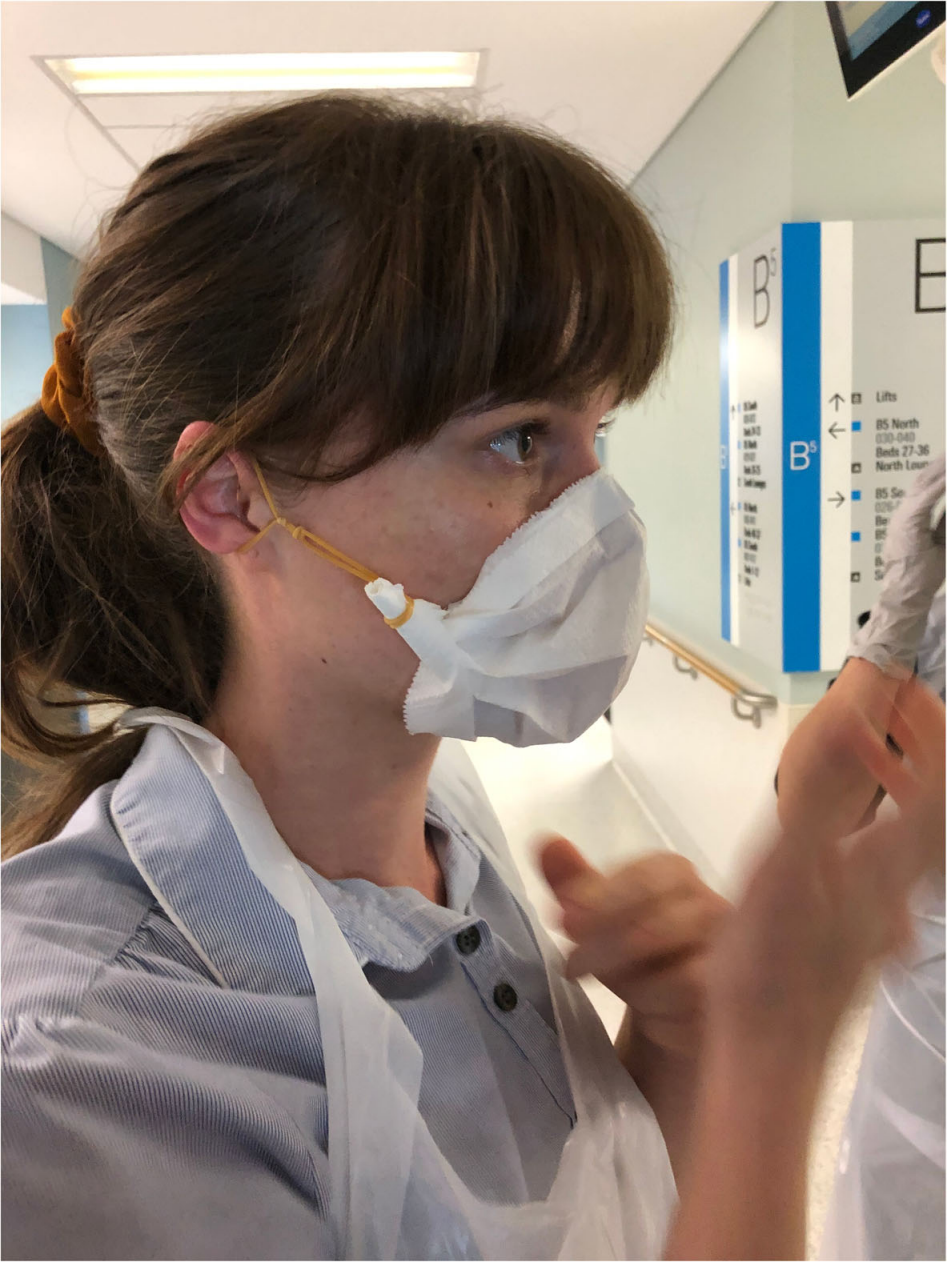
Simulated PPE

**Fig. 6:**
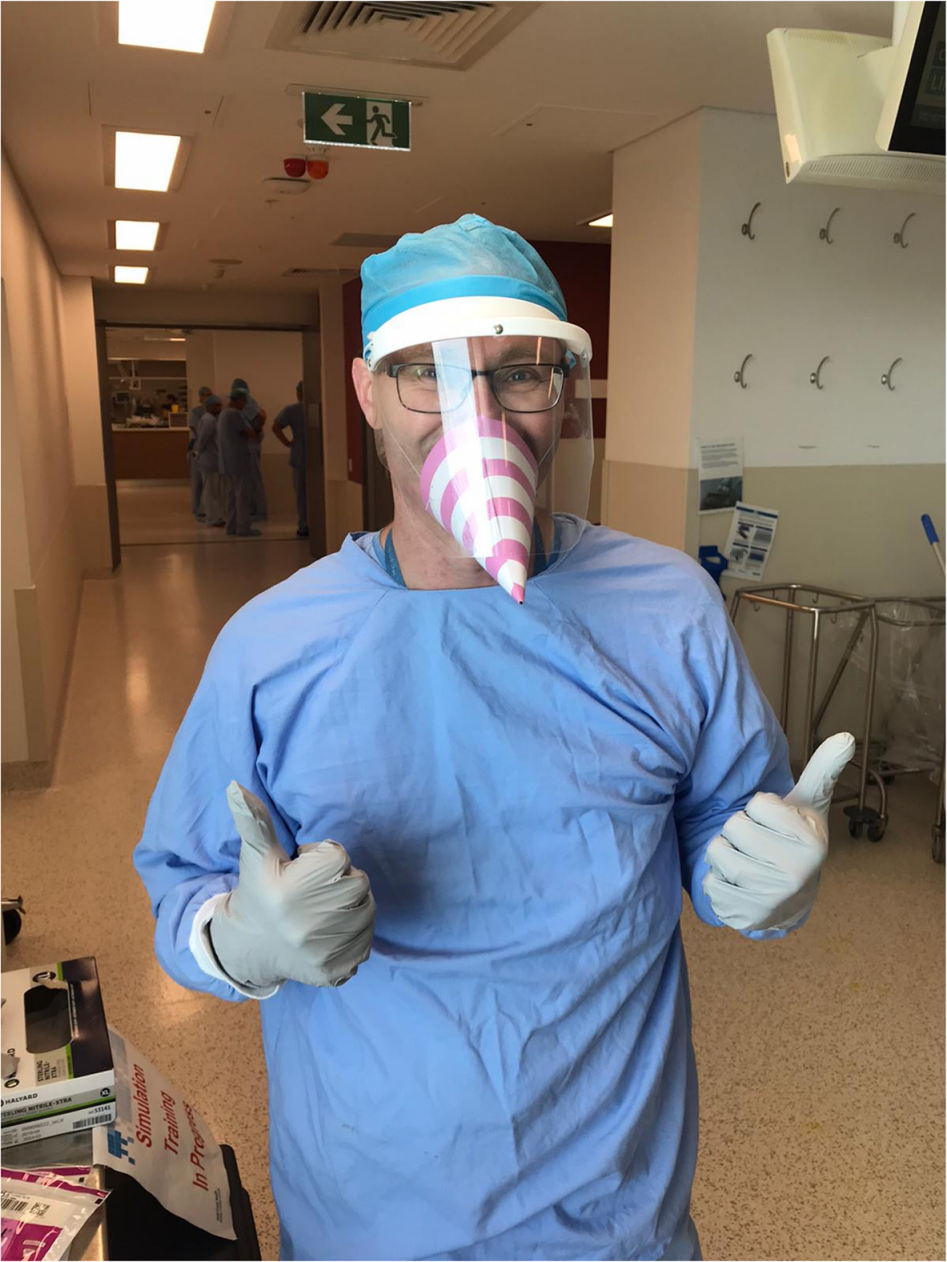
Simulated PPE

The nature of the COVID-19 infection presented specific challenges to conducting in situ simulation with clinical teams. These included the need for physical distancing in pre-briefs and debriefs (Fig. 4), inability to use real PPE in simulation to conserve stocks and the possibility of manikins and other training equipment harbouring viral particles. These practical challenges were addressed in a variety of ways including using simulated PPE (Figs. 5 and 6), video conference enabled debriefs and modifying scenario delivery to be as simple as possible to achieve the objective of the simulation session. We quickly became aware of the power of a simulation session to lessen or exacerbate team anxiety and emphasised the need for short but clear pre-briefings and debriefings.

More traditional challenges to embedding simulation delivery—e.g. shift work, clinician engagement—were lessened with the intense motivation of staff during this period but not absent. We were flexible in targeting night shift staff and identifying champions within specific subgroups, e.g. surgeons.

A translational approach requires a suite of techniques, not limited to in situ simulation [1] and including focused skills training (including lab-based simulation), table top exercises and instructional videos. Mental rehearsal is a valuable simulation tool [16], especially when PPE cannot be spared, and for practising new procedural task sequences (e.g. COVID airway management).

### Build a strong community of practice of simulation educators throughout the institution—to share techniques and maximise simulation delivery capacity during a crisis

Delivering a year’s volume of simulation in 1 month was only possible through a leveraged approach involving our network of ‘clinician simulationists’ with adequate skills and a translational simulation perspective to be semi-autonomous. Opportunities were taken to share generalisable findings and lessons between units (e.g. the challenges of airway management in PPE or the transit of patients from different areas to and within the operating theatre).

Despite our enormous increase in activity, we were still unable to provide the volume of training required to train individuals and teams to a level of proficiency in new processes.

### Advocate for a translational simulation service within the health service to enable rapid responsiveness to a crisis

Carefully targeted and effectively delivered simulation activity cannot be just ‘tuned on’ in the face of a crisis. Our experience has illustrated the need for health services to have a fully integrated, resourced and skilled translational simulation service that can quickly respond to health service needs in a rapidly unfolding crisis. COVID-19 preparation has been a ‘time to shine’ [17] for healthcare simulation, and simulation leaders should be showcasing their work, demonstrating value and thoughtfully reflecting with other health service leaders about how to best approach future challenges.

## Conclusion

Our experience with using simulation for COVID-19 pandemic preparation has sharped reflection on the role of simulation in health service performance and change management, albeit in a unique and urgent context. We encourage simulation leaders to embrace this unique opportunity to innovate and to advocate for healthcare simulation as an integral component of healthcare delivery.

## Supplementary information

**Additional file 1.** Cath lab report COVID.

**Additional file 2.** COVID intubation skills anaesthetics.

**Additional file 3.** COVID MID CT.

**Additional file 4.** COVID PPH Theatre management.

## Data Availability

The datasets used and/or analysed during the current study are available from the corresponding author on reasonable request.

## Abbreviations

COVID-19: Coronavirus disease 2019
PPE: Personal protective equipment
GCHHS: Gold Coast Hospital and Health Service
MET: Medical Emergency Team
