# Supplementary material for: Translational simulation for rapid transformation of health services, using the example of the COVID-19 pandemic preparation
Source: Adv Simul (Lond). 2020 Jun 3;5:9. doi: 10.1186/s41077-020-00127-z (PMC7267758; doi:10.1186/s41077-020-00127-z)
Supplement: Supplementary file 1 — Additional file 1. Cath lab report COVID. [file 41077_2020_127_MOESM1_ESM.pdf]

CCS COVID MET CALL RESPONSE

Simulation 17th March 2020

Scenario Overview: Urgent STEMI arrives from Emergency Department - COVID positive
The patient deteriorates - VF arrest requiring MET Team response (requires intubation)
Responders must don appropriate radiation and PPE protection prior to managing deterioration

Issues Identified

EQUIPMENT

- Inconsistency of PPE worn
- CCS stretched waiting for MET Call Team response - 4 mins. Delays include difficulty finding appropriate fitting lead and donning of PPE
- No available PPE for MET Team (Currently not enough PPE)
- MET Trolley located in the room, defibrillator in material bag (infection control issues)
- CCS Suit requires 2 hour turn around re: cleaning and restocking -may delay
- emergency/routine procedures

STAFF

- No Role allocation for "spotter: No one prioritising of who should enter and don first - No crowd control
- Radiographer and required to enter room and then return to control room to operate imagining equipment
- After hours minimal staff for spotter role
- CCS + MET Call team may be stood down if not using appropriate PPE
- Need to reconsider roles of wardies - not all for CPR
- Unable to identify 'who's who"

Considerations for New Processes/Workflows

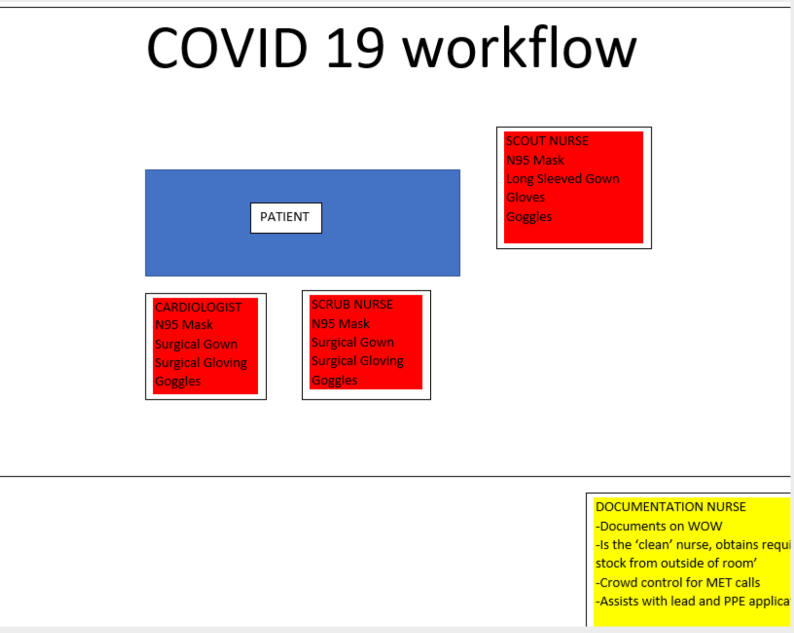

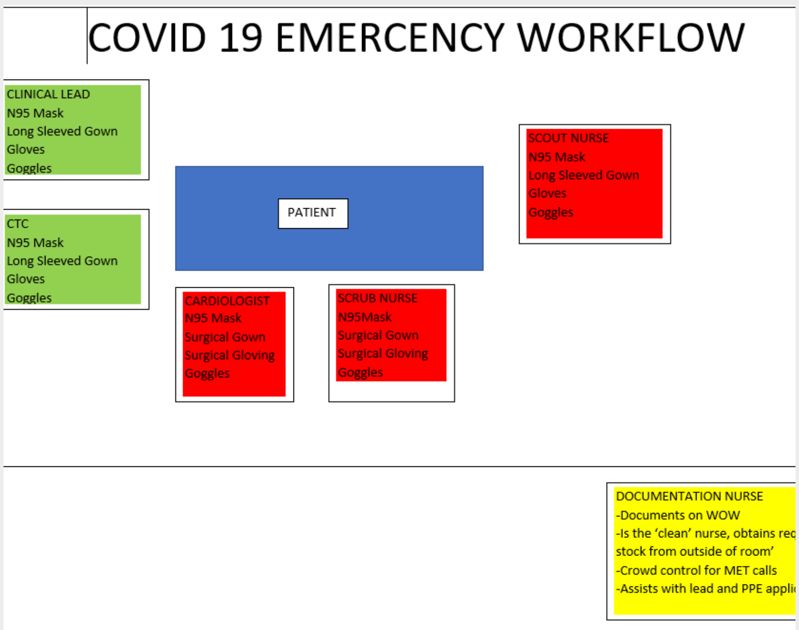

EQUIPMENT

- COVID Dedicated CCS Suite
- Appropriate + adequate PPE
- COVID allocated Leads sizes easily identified
- Defibrillation Bag removed
- Emergency Medications Grab bag/scaled back and MET Trolley outside of room
- Minimise ALL unnecessary equipment form entering room)

STAFF

- Dedicated Spotter/crowd control (who?)
- Prioritise CTC Clinical Lead to enter room first
- Consider the control room as dirty due to Radiographers tasks which need to be completed for patient safety, therefore keep "clean"observers out of control room

GCHHS Gold Cost Sim Service/AHCU/CCS Collaborative Report, March 2020
