# Supplementary material for: Translational simulation for rapid transformation of health services, using the example of the COVID-19 pandemic preparation
Source: Adv Simul (Lond). 2020 Jun 3;5:9. doi: 10.1186/s41077-020-00127-z (PMC7267758; doi:10.1186/s41077-020-00127-z)
Supplement: Supplementary file 2 — Additional file 2. COVID intubation skills anaesthetics. [file 41077_2020_127_MOESM2_ESM.pdf]

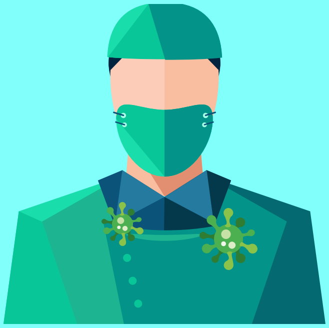

OT COVID 19 Intubation Skills

Practice and Diagnose
COVID 19 Specific Intubation Requirements

16th March, 2020

AIM of Simulation: to practice safe intubation techniques for anaesthetic teams in a COVID positive, otherwise well patient requiring urgent surgery

During the initial phases of response (Containment Phase) it is hoped that by identifying changes to the way intubation is performed in Covid19 cases Anaesthetic teams will be more effectively equipped to safely manage this patient cohort when they arrive for surgery in the Operating Theatre,

Scenario Overview:
A young female that has tested COVID positive requires urgent surgical intervention for acute appendicitis. Anaesthetic teams will be expected to plan and manage an uncomplicated intubation in light of the increased risk of exposure to viral particulates.

Summary of Challenges

Poor Knowledge PPE requirements
Variable skill PPE DONing & DOFFing
Lack of equipment & medications available inside OT
Poor Communication between OT team & Anaesthetic Scout in Anaes Room
Limited staff in OT

Equipment Considerations

PLAN Ahead
Only Take in what you need
LEAVE
Anaesthetic Drug Trolley
Extra Airway Equipment to be managed by Anaes Scout Nurse in Anaes Bay

Infection Control Considerations

Consider prioritising who needs to be in the room.
Control the flow of people in and out of the Theatre
ALL equipment needs to be cleaned prior to removal.
When taking off PPE .....**SLOW DOWN** and follow correct procedure

Opportunities

Formalise the PPE Process specific to Theatre - add in step for double gloving
Create Airway Grab Bag with key equipment for Intubation
Bronchospasm Grab Bag with early treatment medications
Cognitive aid for COVID intubation
Explore options for inter-room communications

Gold Coast Sim Service & Anaesthetic Team Collaboration 2020
