# Supplementary material for: Translational simulation for rapid transformation of health services, using the example of the COVID-19 pandemic preparation
Source: Adv Simul (Lond). 2020 Jun 3;5:9. doi: 10.1186/s41077-020-00127-z (PMC7267758; doi:10.1186/s41077-020-00127-z)

MID CT COVID-19 MET Call Response

Simulation 20th March 2020

1 Scenario Overview

- COVID-19 positive pt requires a CTPA
- The patient will shows signs of respiratory deterioration
- MET Team response with need to manage patient's condition whilst adhering to COVID+ precautions/guidelines

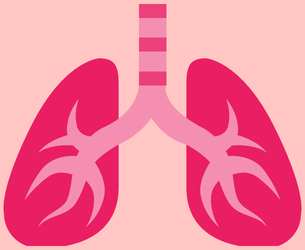

2 Staff/Interfaces

- 8 staff members in the room responding to pt. deterioration
- Difficult to know "who's who" with PPE insitu (mask goggles)
- Unclear communication who should come in the room
- Multiple entry point to CT rooms difficult to police staff entering

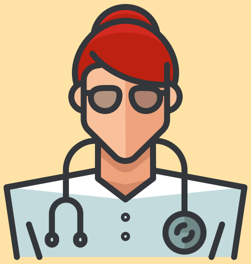

3 Equipment/Resources

- Good use of medication + ventilation grab bags - red MET Trolley outside room with PPE trolley
- No hand rub/bin inside room for doffing
- moved pt onto own bed to manage safely in upright position

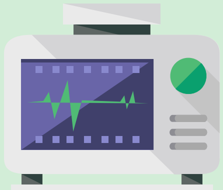

4 Considerations/Opportunities

- Reduce the number of staff in the room
- Write your name on PPE or introduce yourself to team
- Nurse Team leader - "spotter" human/equipment resource management
- Consider how to reduce entry points for MET Call response

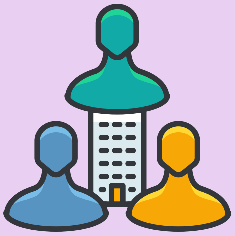

Supplement: Supplementary file 3 — Additional file 3. COVID MID CT. [file 41077_2020_127_MOESM3_ESM.pdf]
