# Supplementary material for: Translational simulation for rapid transformation of health services, using the example of the COVID-19 pandemic preparation
Source: Adv Simul (Lond). 2020 Jun 3;5:9. doi: 10.1186/s41077-020-00127-z (PMC7267758; doi:10.1186/s41077-020-00127-z)
Supplement: Supplementary file 4 — Additional file 4. COVID PPH Theatre management. [file 41077_2020_127_MOESM4_ESM.pdf]

GCUH Covid-19 Maternity

Post Partum Haemorrhage - Theatre Management

Early activation of COVID Obstetric Haemorrhage Alert

OT need more time to organise COVID theatre setup – active this routinely and early

COVID positive status MUST be clearly stated to switch when activating the alert or respond

This ensures the correct COVID dedicated OT is setup

COVID-19

If PPH continues and need for OT is identified activate COVID Obstetric Haemorrhage Respond

Verbal consent only for urgent surgical procedures due to COVID contamination with paper documentation

Maternity team must call theatre team leader before any transfer of patient to OT

This ensures the OT team are ready in PPE to receive the patient prior to transfer

Wherever possible the maternity team should wait until operative theatre assistant (wardie) arrives to transfer the patient to theatre

Once transferred to OT – STOP moment should occur prior to GA

After handover any staff without a n95 PPE needs to leave the OT – PPH is likely to need a GA

After GA - consider the use of a second STOP moment recap once scrub team enter

STOP

Need to consider traditional MTP vs Rotem guided MTP for these patients – as logistics of a Rotem guided MTP may be challenging

Infographic by Dr Belinda Lowe Version 1.2 2.4.20
